# A multi-cancer early detection blood test using machine learning detects early-stage cancers lacking USPSTF-recommended screening

**DOI:** 10.1038/s41698-024-00568-z

**Published:** 2024-04-17

**Authors:** Janet Vittone, David Gill, Alex Goldsmith, Eric A. Klein, Jordan J. Karlitz

**Affiliations:** 1 Mayo Clinic, Rochester, Minnesota USA; 2 Intermountain Healthcare, Salt Lake City, UT USA; 3 Colorado Center of Medical Excellence, Denver, CO USA; 4 GRAIL, LLC, Menlo Park, CA USA

**Keywords:** Cancer screening, Next-generation sequencing

## Abstract

US Preventive Services Task Force (USPSTF) guidelines recommend single-cancer screening for select cancers (e.g., breast, cervical, colorectal, lung). Advances in genome sequencing and machine learning have facilitated the development of blood-based multi-cancer early detection (MCED) tests intended to complement single-cancer screening. MCED tests can interrogate circulating cell-free DNA to detect a shared cancer signal across multiple tumor types. We report real-world experience with an MCED test that detected cancer signals in three individuals subsequently diagnosed with cancers of the ovary, kidney, and head/neck that lack USPSTF-recommended screening. These cases illustrate the potential of MCED tests to detect early-stage cancers amenable to cure.

## Introduction

Only select cancers (e.g., breast, cervical, colorectal, and lung) have United States Preventive Services Task Force (USPSTF)-recommended screening guidelines^1^. Although the use of these single-cancer screening tests has reduced cancer-related mortality for these malignancies, ~70% of deaths due to cancer in the U.S. among those 50–79 years of age are caused by cancers without USPSTF-recommended screening. Partly as a result, US cancer mortality exceeds 600,000 cases/year^2^.

**Fig. 1:**
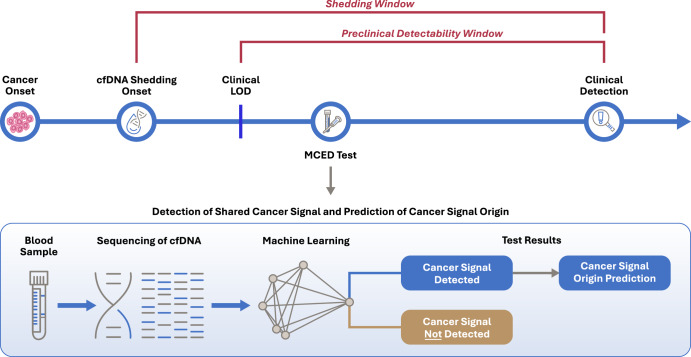
The MCED paradigm. cfDNA cell-free DNA, LOD limit of detection, MCED multi-cancer early detection.

A recently developed multi-cancer early detection (MCED) test (Galleri®, GRAIL, LLC, Menlo Park, CA), intended to complement USPSTF-recommended screening is clinically available as a laboratory-developed test (LDT) performed in GRAIL’s clinical laboratory with accreditations from the College of American Pathologists (CAP) and certification under Clinical Laboratory Improvement Amendments (CLIA)^3^. This MCED test comprises a single blood draw followed by a targeted methylation assay of cell-free DNA (cfDNA) and a machine learning-based algorithm to detect a shared cancer signal across multiple cancer types^4^. The test capitalizes on a preclinical detection window created by tumor shedding of cfDNA (Fig. 1) with results provided within 10 working days of sample receipt. The test report indicates whether a cancer signal was detected, and if so, provides up to two predictions, ranked by signal strength, for the likely organ or organ system of cancer involvement (i.e., cancer signal origin or CSO) out of 21 pre-specified options, allowing for targeted diagnostic evaluations^5^. The MCED platform supporting the test has been extensively studied with >385,000 participants having completed or currently enrolled/enrolling in clinical studies.

This MCED test was developed and validated in the Circulating Cell-Free Genome Atlas study (CCGA; NCT02889978, *N* = 15,254)^4,6,7^, a case-control study in which it detected a cancer signal across >50 cancer types with a low false positive rate (specificity of 99.5%) and predicted the CSO with approximately 90% accuracy^7^. In CCGA substudy 1, which focused on the discovery and selection of the highest performing assays, whole genome bisulfite sequencing outperformed targeted and whole genome sequencing approaches and a methylation-based assay was selected for further development in the subsequent substudies. The overall sensitivity for cancer detection was 51.5%, which varied by cancer type and stage^7,8^. Stage I-III sensitivity in 12 pre-specified cancers that account for nearly two-thirds of annual cancer deaths in the US was 67.6% (95% confidence interval: 64.4% to 70.6%)^7^. Cancers included in this subgroup were pancreatic, ovarian, head and neck, and multiple other aggressive cancer types that do not have USPSTF-recommended screening guidelines. The positive predictive value (PPV) reported from that study was 44%, which is an order of magnitude higher than most single-cancer screening tests (i.e. mammography, CT chest, FIT, and others)^9–11^.

A prospective single-arm return of results study using this MCED platform in adults >50 years without signs or symptoms of cancer (PATHFINDER; NCT04241796; *N* = 6662) demonstrated implementation feasibility with a first or second CSO prediction accuracy of 97%^12^. Approximately half (48%) of those with a non-recurrent cancer were diagnosed at an early stage (stage I or II), and more than 70% of cancers diagnosed were cancers that do not have USPSTF-recommended screening guidelines. In this study, which took place during the height of the COVID-19 pandemic, 73% of true positives received diagnostic resolution within 3 months and 50% within 2 months or less^12^.

This MCED test is intended to complement and not replace USPSTF-recommended screening, with two key goals: finding early-stage cancers when a cure is most likely and increasing the overall cancer detection rate by enabling the detection of cancers that currently lack screening options. Clinical implementation of the MCED test in a population will detect cancers with a range of stages, including early and late-stage disease.

This multi-cancer screening approach represents a new paradigm^13^, and clinical data on its real-world performance are still accruing. Findings from approximately 53,000 people who underwent community-based MCED testing with Galleri have recently been reported and show a 0.95% cancer signal detection rate, which is in line with the cancer incidence expected from the Surveillance, Epidemiology, and End Results (SEER) Program^14^. The test was able to detect a cancer signal from cancer types that lack screening tests, including cancers at early stages. The MCED test accurately predicted the origin of the cancer signal in 91% of cases. Follow-up of cases with a cancer signal detected (CSD) is currently ongoing through a quality assurance program, which will allow for detailed reporting of these real-world outcomes. Here, we report three specific clinical cases of early-stage solid organ cancers detected by the Galleri MCED test as an illustration of its potential to (1) detect early-stage cancers; (2) detect cancers that lack USPSTF-recommended or other screening guidelines; and (3) direct diagnostic evaluation based on CSO predictions.

## Results

### Case 1: Stage I ovarian cancer

**Fig. 2:**
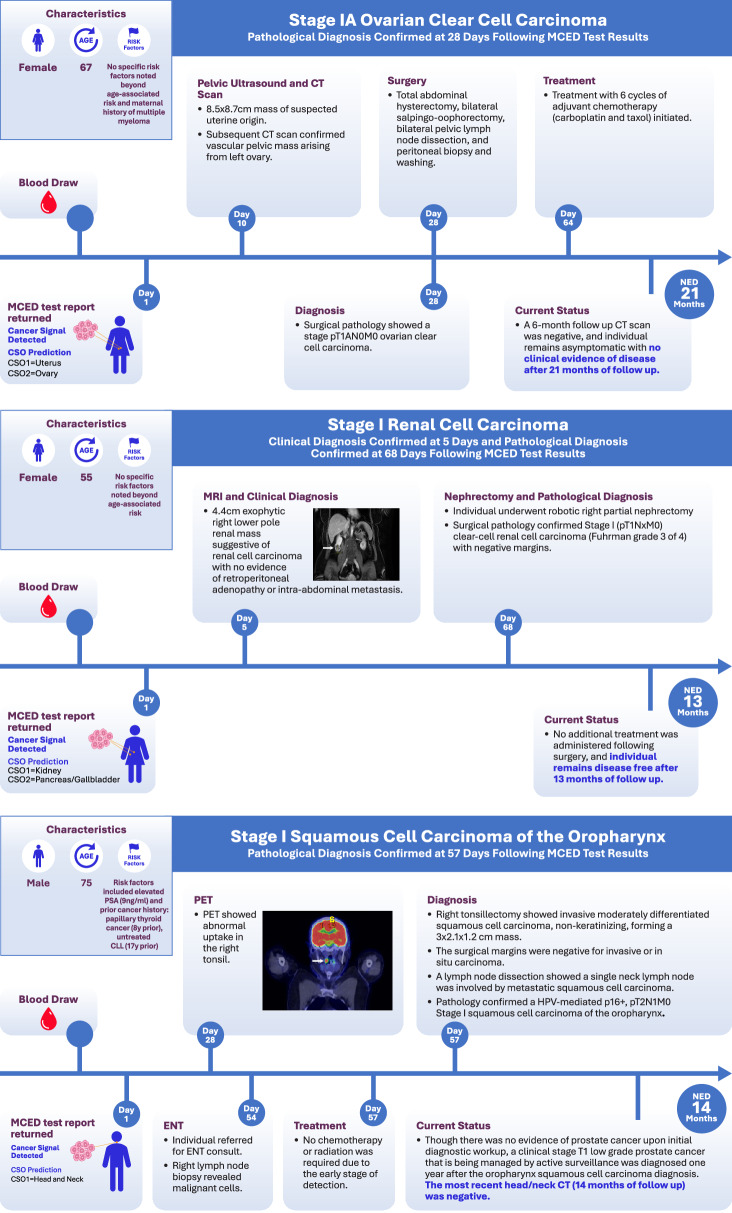
Diagnostic and treatment decisions in three cases of early cancer detection with the MCED test. CSO cancer signal origin, CT computed tomography, ENT ear, nose, and throat, MCED multi-cancer early detection, MRI magnetic resonance imaging, NED no evidence of disease, PET positron emission tomography.

An asymptomatic 67-year-old woman was tested at her request as part of a routine physical. She was on no medications and had no significant prior medical history, though her mother had a history of multiple myeloma. The MCED test returned a CSD result with CSO predictions of the uterus (first prediction) and ovary (second prediction) (Fig. 2). Diagnostic evaluation included a pelvic ultrasound showing an 8.5 cm × 8.7 cm heterogeneous pelvic mass followed by a CT scan confirming a vascular pelvic mass arising from the left ovary. Twenty-eight days after the test, the individual underwent total abdominal hysterectomy, bilateral salpingo-oophorectomy, bilateral pelvic lymph node dissection, and peritoneal biopsy and washing. Surgical pathology demonstrated AJCC stage 1A high-grade ovarian clear-cell carcinoma without lymph node involvement. The individual was subsequently treated with 6 cycles of adjuvant chemotherapy (carboplatin and taxol), consistent with National Comprehensive Cancer Network (NCCN) guidelines for high-grade stage pT1AN0M0 (stage I) ovarian cancer. The time to diagnostic resolution, defined as the interval between the day MCED test results were reported to final pathology, was 28 days. A 6-month follow-up CT scan was negative. The individual is symptom-free and with no evidence of disease (NED) as of 21 months of follow-up.

### Case 2: Stage I renal cell carcinoma (RCC)

An asymptomatic non-smoking 55-year-old woman with no known cancer risk factors beyond age requested the MCED test as a covered benefit within her healthcare system. A CSD result was returned with CSO predictions of kidney (first) and pancreas/gallbladder (second**;** Fig. 2). An abdominal MRI performed 5 days later showed a 4.4 cm exophytic right lower pole renal mass suggestive of RCC with no evidence of retroperitoneal adenopathy or intra-abdominal metastasis or other lesions (Fig. 2). Diagnostic resolution was achieved on day 68, with complete excision achieved by partial nephrectomy and pathology demonstrating Fuhrman grade 3 (out of 4) stage I clear-cell RCC with negative margins. Consistent with NCCN guidelines for pT1NxM0 RCC, no additional therapy was given. The individual remains asymptomatic and NED at 13 months of follow-up.

### Case 3: Stage I squamous cell carcinoma (SCC) of the oropharynx

An asymptomatic 75-year-old non-smoking man with no known history of human papillomavirus (HPV) infection had an MCED test at his request. His medical history included a papillary thyroid cancer treated with radiation therapy 8 years prior, untreated chronic lymphocytic leukemia (Rai stage 0; 13q, CD38, and ZAP-70 negative) 17 years prior, and a current prostate-specific antigen (PSA) value of 9 ng/ml (normal <4 ng/ml). A CSD result was returned with the single CSO of head and neck (Fig. 2). Given the individual’s previous malignancies, increased risk due to previous radiation therapy, an elevated PSA level, and a CSD result with the MCED test, a PET-CT scan was performed, which showed abnormal uptake in the right tonsil. The individual was referred to an otolaryngologist and underwent a right cervical lymph node biopsy that showed the presence of malignant cells. A tonsillectomy revealed a moderately differentiated non-keratinizing SCC measuring 3.0 × 2.1 ×1.2 cm with negative surgical margins. One of the 27 cervical lymph nodes showed metastatic SCC. Pathology confirmed an HPV-mediated p16+, pT2N1M0 (stage I) SCC of the oropharynx^15^. Diagnostic resolution was achieved in 57 days following the test. Consistent with NCCN guidelines for pT2N1M0 p16+ disease, no additional therapy was given. There was no evidence of prostate cancer upon initial diagnostic workup, though a clinical-stage T1 low-grade prostate cancer that is being managed by active surveillance was diagnosed one year after the oropharynx SCC diagnosis. With respect to that SCC diagnosis, the most recent head/neck CT (14 months follow-up) was negative.

## Discussion

We report early real-world experience with a validated MCED test designed for cancer screening and available for clinical implementation. Although the MCED test detects cancer across all stages, we describe the early detection (Stage I) of 3 cancers that are not covered by USPSTF-recommended screening or other routine screening tests in individuals without known risk factors. All 3 individuals were asymptomatic and thus unlikely to be otherwise diagnosed at an early stage, and 2 of them had no risk factors other than age. In all 3 cases, the CSOs were proven correct by pathology and helped guide efficient diagnostic evaluation. Diagnostic resolution ranged from 28–68 days, consistent with that reported in the PATHFINDER study^12^. All three were eligible for and underwent curative-intent treatment with guideline-concordant care.

The experiences of these individuals highlight this technology’s potential to detect early-stage cancers in asymptomatic individuals and illustrate the ability of CSO prediction capability to achieve diagnoses efficiently. With respect to the specific cancers discussed here, ovarian cancer tends to present at a late-stage^16^, is challenging to diagnose due to non-specific or absent symptoms^17^, and screening is mainly considered in a subset of individuals at genetically high risk, with marked limitations in screening modality performance (i.e., transvaginal ultrasound)^18–20^. Similarly, early-stage RCCs are usually asymptomatic and are generally detected incidentally on imaging with a notable potential for overdiagnosis^21^. In the case described here, the individual had a higher grade histology, which is predictive of more aggressive behavior. There are no current recommendations for RCC screening in individuals at average risk. Finally, no routine screening programs or tests exist for oropharyngeal cancers beyond findings noted through routine oral exams in dental offices or self exams^15^. Although HPV is a risk factor for oropharyngeal cancers, there is no approved HPV screening test for the throat, in contrast to cervical cancer^15^. Additionally, the detection of less common cancers such as oropharyngeal cancer in the real-world is particularly notable, given that it may not be viable to have single-cancer screening tests for less common cancers.

In the absence of screening, an undetected early-stage cancer can progress to a more advanced stage before the presentation of clinical symptoms that would lead to a diagnosis, by which point the prognosis may have become less favorable. The stage dependency for survival outcomes for these cancer types suggests that these three cases are likely to have favorable long-term outcomes (survival outcomes for these cancers at localized, regional, and distant stages, respectively, are: ovarian, 93.1%, 74.2%, and 30.8%; RCC, 93.3%, 74.7%, and 15.7%; and oropharyngeal SCC, 83.1%, 77.8%, and 48.7%)^22,23^.

The technology underlying MCED tests relies on the detection of tumor-associated circulating cfDNA. As such, not all cancers are detectable with this technology as not all tumors and tumor types shed cfDNA in quantities above the clinical limit of detection (LOD). For example, in the CCGA study, whereas overall sensitivity for head and neck cancer was 85.7% and ovarian cancer 83.1%, it was only 18.2% for renal cancer, which is known to be among the lower shedding tumor types. For low-grade prostate cancers, which are associated with indolent behavior, detection rates are under 4%^7^. The three cases described here benefited from the fact that their tumors shed cfDNA at a level above the clinical LOD for this test; several lines of evidence suggest that tumors that do so are associated with the potential for aggressive behavior even in early stages^24,25^.

Some considerations should be noted when weighing the clinical insights supported by these cases. First, in these 3 cases, CSO calls corresponded with the tumor type diagnosed. There was no evidence of a second type of cancer during comprehensive clinical evaluation and follow-up for at least one year in all 3 cases, indicating that tumor shedding was from the cancers that were diagnosed and treated. In addition, though the individual cases presented here are by design meant to serve as case illustrations, it is still appropriate to acknowledge that they represent only a small subset of a larger set of individuals who have received this test.

MCED represents a new paradigm with the potential to address a significant unmet need in cancer screening. By combining next-generation genome sequencing and machine learning, MCED tests can detect multiple cancer types, including those that are insufficiently prevalent to allow for efficient single-cancer screening^26,27^. Because this test detects a shared cancer signal across multiple cancer types, individual cancer prevalence can be aggregated across multiple cancers to improve screening efficiency, resulting in a much higher PPV and overall cancer detection rate than currently endorsed screening tests^2,28^. In addition, the machine-learning algorithms continuously learn from new data of the kind presented here, so the test performance characteristics can continuously improve.

Machine learning is a subcategory of the broader field of artificial intelligence and uses algorithms to automatically learn insights and recognize patterns from data, applying that learning to make increasingly better decisions^29^. In this case, to learn which cfDNA fragments may have originated from cancerous cells, the classifier algorithm was initially trained on sequencing data from more than 15,000 individuals in the CCGA study that enrolled participants between 2016 and 2018^6,7^. This study comprised 6670 individuals without cancer and 8584 individuals with cancer for whom the cancer type was also recorded along with any comorbidities. The first step of the classifier training phase was deciding the right way to encode DNA methylation status so that it is computer-readable (“representation”). Second, the algorithm compared the patterns of methylation from individuals without cancer in CCGA to the individuals known to have cancer and derived a shared cancer signal (“learning”). This cancer signature is almost never observed in people known not to have cancer. Finally, the algorithm assigned a score to each individual that estimated the likelihood that they had cancer, and then assigned each of these likelihoods to one of two bins: cancer signal detected, i.e., test positive, or not, i.e., test negative (“thresholding and scoring”). Once the classifier was trained in this way and passed the representation, learning, and scoring stages, it was tested and validated on additional data that it had not seen yet. If the classifier returns a test positive, a second algorithm is triggered, to learn which cells the cancerous cfDNA fragments came from, resulting in the prediction of a CSO. The training stage runs on 1600 computer processors and takes four hours, while the day-to-day predictions run on 48 processors and take one minute. This approach was selected as it enables a continuous learning environment, where we can train the classifier on more diverse data driving improved performance over time.

Unlike current single-cancer tests, which are calibrated to maximize sensitivity and thus have higher false positive rates, MCED tests are designed for high specificity and very low false positive rates (<1%) with promise to minimize potential harms. Importantly, the MCED test used in these cases provides a prediction of the cancer signal origin, which can facilitate streamlined diagnostic evaluations. These 3 cases are not meant to stand on their own as evidence for clinical use but provide examples of the power and potential of the test for early-stage diagnosis and how new AI-based technologies can be directly applied to real-world clinical settings to optimize patient care. The cases should be reviewed in the context of robust clinical trial data and ongoing real-world evidence accrual, which support clinical use as an LDT. When used at a population level, MCED tests have the potential to reduce cancer mortality by intercepting cancers at earlier stages^28^.

## Methods

Cases were selected from an initial ~500 individuals (out of a total of ~53,000 tests) with a CSD result from 04/20/2021 (date of commercial availability) to 12/31/2022. Individuals were required to have met the basic criteria for the intended use population (i.e., >50 years and without clinical suspicion of cancer), have a complete health record available for review, and were diagnosed with cancer types that lack USPSTF-recommended screening. These cases also illustrate the utility of CSO predictions to guide diagnostic evaluation. Tests were ordered by their primary care physicians in private practice (A. Goldsmith) or a large healthcare system (D. Gill, J. Vittone). Ordering physicians complied with all relevant ethical regulations in patient interactions, in line with ethical norms and standards in the Declaration of Helsinki. This limited dataset was exempt from formal IRB review, and the individuals whose cases are shared here gave informed, written consent to be included within a piece of the published literature.

### Reporting summary

Further information on research design is available in the Nature Research Reporting Summary linked to this article.

### Supplementary information

REPORTING SUMMARY

## Data Availability

All known and relevant data for the three cases have been shared in the manuscript.
